# 94 One-size-fits-all? Customizing a Standardized Onboarding Approach to Improve New Graduate Nurses’ Learning Opportunities

**DOI:** 10.1093/jbcr/iraf019.094

**Published:** 2025-04-01

**Authors:** Emily Snyder

**Affiliations:** Medical City Plano

## Abstract

**Introduction:**

Nurses who recently came off of orientation were voicing frustrations and concerns when caring for a burn patient due to their lack of exposure during orientation. When reviewing past patient assignments for these nurses, it was discovered that many nurses were having very little experience with the burn population compared to the trauma population prior to completing orientation.

**Methods:**

This retrospective review utilized the Burn Trauma Intensive Care Unit’s (BTICU) patient logbook and a new created weekly update form completed by the new graduate nurses. The goal was to ensure that while on orientation, the nurses cared for a burn patient 25%-30% of the time, closely mirroring the ratio they would see once they were practicing independently.

The Burn Educator (BE) reviewed the patient assignments for the nurses on orientation during the Fall 2022 and Spring 2023 cohorts via the BTICU patient logbook. During their orientation, the Fall cohort cared for a burn patient 6.9% of the time and the Spring cohort cared for a burn patient 14.4% of the time. This did not give them enough exposure to the burn population to build confidence or represent the ratio of burn to trauma patients they would care for once on their own.

Starting with the next two cohorts the BE tracked their patient assignments in real time and reported the percentages back to the nurse manager. The nurses in this cohort cared for a burn patient 32% of the time. Even though their exposure rate went up, these nurses tended to get assigned the same patients on a routine basis.

In an attempt to create a more robust orientation experience, the next two cohorts were required to fill out a weekly update form with their preceptors. These forms provided sections to complete a brief patient summary, weekly goals, and preceptor comments. The BE then analyzed these forms and patient assignments on a weekly basis and sent feedback back to the manager regarding the types of assignments the nurses would need the following week. Both of these cohorts cared for a burn patient 30% of the time. These forms enabled the nurse to see a variety of burn and trauma patients and create better learning opportunities for the nurse while on orientation.

**Results:**

After the implementation of patient assignment monitoring and the weekly update form, the number of burn patients cared for during orientation significantly increased. Nurses on orientation were being assigned patients that matched their needs and were able to experience a greater variety of patients in both the burn and trauma population.

**Conclusions:**

Real time monitoring of patient assignments is one way to create a more robust experience for nurses onboarding.

**Applicability of Research to Practice:**

Further research is needed to see if customizing an orientation plan will help reduce the rate of burnout in new graduate nurses.

**Funding for the Study:**

